# Adrenomedullin Function in Vascular Endothelial Cells: Insights from Genetic Mouse Models

**DOI:** 10.2174/157340211799304761

**Published:** 2011-12

**Authors:** Natalie O Karpinich, Samantha L Hoopes, Daniel O Kechele, Patricia M Lenhart, Kathleen M Caron

**Affiliations:** 1 Department of Cell and Molecular Physiology, The University of North Carolina at Chapel Hill, Chapel Hill, North Carolina, USA; 2 Department of Genetics, The University of North Carolina at Chapel Hill, Chapel Hill, North Carolina, USA

**Keywords:** Adrenomedullin, angiogenesis, endothelial, lymphangiogenesis, mouse model, permeability, CLR, RAMPs.

## Abstract

Adrenomedullin is a highly conserved peptide implicated in a variety of physiological processes ranging from pregnancy and embryonic development to tumor progression. This review highlights past and present studies that have contributed to our current appreciation of the important roles adrenomedullin plays in both normal and disease conditions. We provide a particular emphasis on the functions of adrenomedullin in vascular endothelial cells and how experimental approaches in genetic mouse models have helped to drive the field forward.

## INTRODUCTION

### The Multifunctional Adrenomedullin Peptide

**Fig. (1):**
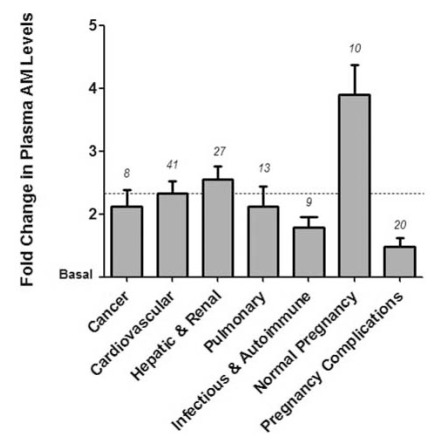
**Fold Change in Plasma Adrenomedullin Levels in a Variety of Human Conditions.** Bars indicate average fold change in circulating AM levels in various disease categories or conditions based on published human clinical data. The dashed horizontal line at 2.33 represents the average fold increase in plasma AM levels across all conditions depicted. Number above each bar indicates the number of published observations assessing plasma AM levels in each category. The clinical papers that were used for our analysis are listed according to the following broad categories: cancer [117- 122], cardiovascular [4, 117, 119, 123-150], hepatic and renal [130, 131, 133, 134, 151-162], pulmonary [6, 131, 163-168], infectious & autoimmune [169-175], normal pregnancy [63, 64, 66-67, 176-180], and pregnancy complications [63, 67, 180-192].

Adrenomedullin (gene=*Adm*; protein=AM) is a highly conserved multifunctional peptide that is implicated in a wide variety of physiological processes including angiogenesis and cardiovascular homeostasis [1]. For over a decade, the association of ~2-fold elevations in plasma levels of AM peptide with a wide variety of cardiovascular disease conditions has prompted intense inquiry into understanding the functions and roles of AM in human disease (Fig. **1**). Moreover, the recent development of highly precise methods for the quantitation of midregional proadrenomedullin (MR-proADM) as a reliable surrogate of mature AM plasma levels [2], has paved the way for the introduction of AM as a clinically useful biomarker for the staging of adverse cardiovascular events, including myocardial infarction, sepsis and community acquired pneumonia [3-6]. While it is clear that AM can elicit powerful effects on vascular smooth muscle cells and thus acutely modulate vascular tone, numerous studies in the past 5 years have elucidated essential functions of AM on vascular endothelial cells. In the following sections we review the multi-faceted role of AM in endothelial cells during development, consider how perturbations in AM signaling may lead to vascular pathologies, and highlight recent discoveries regarding AM that have contributed in substantial ways to the broader field of vascular biology. Many of these discoveries have been unraveled through the use of sophisticated genetic animal models (Tables **1** and **2**), and so we have placed a special emphasis on describing the merits and shortcomings of these approaches and also highlighting current questions that are of predominant interest to the field today.

### Adrenomedullin GPCR-Mediated Signaling in Endothelial Cells

G-protein coupled receptors (GPCRs) are widely expressed proteins that span the cell membrane 7 times and respond to a variety of stimuli including peptides, proteins, small organic compounds, lipids, amino acids, and cations. AM binds and signals through the GPCR calcitonin receptor-like receptor (gene=*Calcrl*; protein=CLR). The discovery of a novel class of GPCR associated proteins called receptor activity-modifying proteins (gene=*Ramp*; protein=RAMP) [7] provided insight into how GPCRs signal. The RAMPs are single-pass transmembrane accessory proteins that regulate the translocation of GPCRs to the plasma membrane as well as provide ligand specificity to these receptors. The tissue specific and temporal expression pattern of RAMPs determines the responsiveness of GPCRs to particular ligands. For example, AM binds to the CLR receptor when CLR is associated with either RAMP2 or RAMP3. However, co-expression of CLR with RAMP1 changes the ligand specificity to another potent vasodilator called calcitonin gene-related peptide (CGRP), a related family member of the AM peptide. The ability of CLR to bind multiple ligands provides a unique mechanism by which the receptor can initiate a variety of signaling pathways. Since the AM receptor CLR and the 3 mammalian RAMPs are highly expressed in the vasculature, this cell signaling paradigm is being intensely investigated to determine how it can be exploited for the potential treatment of conditions such as pulmonary hypertension [8], cardiovascular disorders [9], and the inhibition of cancer metastasis [10].

The binding of AM to its receptor CLR results in a myriad of downstream effects including modulation of endothelial cell survival, proliferation, and vessel permeability. For example, AM-induced proliferation and migration of lymphatic endothelial cells is mediated in part by cAMP and downstream MEK/ERK pathways [11]. Similar results were shown using cultured human umbilical vein endothelial cells (HUVECs). AM-mediated induction of HUVEC proliferation and migration through activation of PKA, PI3K, and focal adhesion kinase were observed and then further substantiated in whole animal studies [12, 13]. AM induced the proliferation and migration of cultured HUVECs [12] and numerous studies have shown a direct role for AM in endothelial growth and survival [14-16].

Using *in vitro* experiments, AM was found to regulate the permeability and migration of HUVECs [17]. Previous studies indicated that adult *Ramp2^+/- ^*mice had increased vascular permeability and overexpression of *Ramp2 *in BECs resulted in reduced permeability [18]. AM also reduces the permeability of HUVECs and pulmonary artery endothelial cells treated with permeabilizing agents including hydrogen peroxide and thrombin [19]. AM has been shown to regulate the transport of molecules across the blood brain barrier in cerebral endothelial cells by modulating permeability [20]. In cerebral endothelial cells, AM regulated various functions of the blood brain barrier including increasing transendothelial electrical resistance, reducing fluid-phase endocytosis, and reducing permeability for sodium fluorescein which indicate that the cerebral endothelial cell junctions are tightened by AM [20]. Also in an *in vivo *model, AM treatment reduced lung vascular permeability resulting from ventilator use in a mouse model where prolonged mechanical ventilation was administered [21]. Overall, these data provide evidence for the role of AM as a junctional tightening factor to help regulate endothelial cell permeability. 

Although AM functions to promote endothelial cell growth and proliferation in both the blood and lymphatic vasculatures, Fritz-Six *et al.* have shown that there is an enhanced effect of AM on lymphatic endothelial cells (LECs) as compared to blood endothelial cells (BECs) [22]. This biological distinction in AM function is based upon the finding that LECs are enriched in the expression of AM and its receptor components, *Calcrl* and *Ramp2* [22-24]. This increase in *Calcrl* expression is mediated in part by induction of the transcriptional regulator of lymphatic specification, *Prox1* [22]. It is therefore not surprising that loss of any component of the AM signaling axis (*Adm*, *Calcrl*, or *Ramp2*) results in embryonic lethality associated with profound lymphatic vascular defects [22]. Furthermore, several *in vitro* and *in vivo* experiments reveal that AM controls lymphatic permeability and flow through reorganization of junctional proteins ZO-1 and an adherens protein VE-Cadherin, independent of changes in junctional protein gene expression [25]. Administration of AM to a monolayer of LECs resulted in tightening of the lymphatic endothelial barrier by reorganization of a tight junction protein at the plasma membrane to form continuous cell-cell contacts. Through the use of *in vivo* tail microlymphography, local administration of AM in a SvEv129/6 mouse tail resulted in decreased velocity of lymph uptake from the interstitial space and movement through the lymphatic dermal capillaries in the tail [25]. Thus, it becomes critically important to consider the pleiotropic effects of AM not just on blood endothelial cells, but also on neighboring lymphatic vessels—a dynamic that may ultimately help resolve the complex functions of AM peptide in cardiovascular disease, tumor progression and inflammation. 

While activation of GPCRs typically leads to induction of classical second messenger signaling systems, it is now appreciated that more complex levels of regulation exist [26, 27]. Therefore, it is not surprising that pathway cross-talk is one mechanism through which AM modulates certain endothelial cell functions. For example, Yurugi-Kobayashi *et al.* describe a novel embryonic stem cell differentiation system to study mechanisms of arterial-venous specification. They demonstrated that coordinated signaling of AM/cAMP, VEGF, and Notch induces arterial endothelial cell differentiation from vascular progenitors [28]. Furthermore, GPCR-induced transactivation of receptor tyrosine kinases is another mechanism that allows interaction between signaling molecules. Evidence exists that AM and VEGF pathways are likely to interact in endothelial cells. Although an earlier study claimed that AM-induced capillary tube formation in HUVECs was independent of VEGF activation [14], a more recent study by Guidolin *et al.* demonstrated that VEGFR2 inactivation inhibited AM-mediated angiogenesis in HUVECs [29]. This latter finding suggests that the pro-angiogenic effects of AM require transactivation of the receptor tyrosine kinase VEGFR2. Although controversy still exists regarding the degree of cooperation between pathways, it is certainly intriguing to consider that regulation of endothelial cell biology may very likely involve coordination of multiple signaling molecules. We now must begin to unravel these complexities and elucidate whether these interactions occur differentially in blood and lymphatic endothelial cells and identify the intermediate molecular players involved in pathway cross-talk in the vasculature.

## DEVELOPMENT

### Endothelial Adrenomedullin Signaling is Essential for Embryonic Development

Work by multiple independent groups has established the importance of AM signaling during development. The use of gene targeted mouse models clearly indicates that functional AM signaling is essential for embryonic survival. The genetic ablation of *Adm* [30-32], *Calcrl* [33], *Ramp2 *[18, 22, 34] or the enzyme responsible for functional AM amidation, *peptidylglycine alpha-amidating monooxygenase *(*PAM*) [35] all result in midgestational lethality associated with severe interstitial edema and cardiovascular defects. The conserved phenotypes between these knockout (KO) mice is compelling genetic evidence that the CLR/RAMP2 complex is the main receptor of AM during development*,* and also is the first *in vivo* confirmation that RAMP2 functionally interacts with CLR [22].

Although the overt phenotypes of these KO mice are conserved, the physiological cause of edema and lethality is still debated. One possible hypothesis is that loss of AM signaling causes developmental cardiac abnormalities that lead to heart failure, thus resulting in edema and death that is similar to previously characterized KO mice with developmental heart failure [36-38]. Supporting this line of thought, our lab showed that *Adm*^-/-^, *Calcrl*^-/-^, and *Ramp2*^-/-^ mice have smaller hearts due to decreased myocyte proliferation and increased apoptosis. Additionally, they have increased left ventricle trabecularization, which leads to decreased ventricular volume [22, 30, 33]. However, an alternative hypothesis is that vascular defects are responsible for the phenotypes, since *Adm* [30], *Calcrl *[33], and *Ramp2 *[18] are abundantly expressed in the developing endothelium and vascular smooth muscle cells (vSMC). To help resolve between the two hypotheses, we generated an endothelial-specific *Calcrl*^-/-^ mouse using a *Tie2* promoter to drive *Cre recombinase* expression which recapitulated the phenotype observed in global KO mice [22], indicating that AM signaling in endothelial cells is essential for embryonic development. A remaining caveat to this conclusion is the fact that Tie2-Cre mediated excision also occurs in developing endocardial cells. Therefore, to definitively determine if cardiac abnormalities contribute to this phenotype the reverse experiment using *Cre* lines specific to cardiac myocytes would be beneficial. 

Although vascular defects are responsible for the edema in these KO mice, it remained unclear whether defects in the blood or lymphatic endothelium were the principle cause of the phenotypes. Given the role of AM in regulating vascular permeability, it seems reasonable that loss of AM signaling could lead to increased vascular permeability and a resulting build up of interstitial fluid. In support of this idea, the KO mice have thinner aorta and carotid artery walls due to a decrease in vSMC proliferation [18, 30, 33], although the endothelium lining the aorta appeared to be normal [33]. There are reported abnormalities in endothelial basement membranes and a down-regulation of junctional proteins in *Adm*^-/-^ and *Ramp2*^-/-^ embryos that may lead to increased vascular permeability and hemorrhage [18, 31], but these phenotypes were observed in a small proportion of animals and not conserved in all studies. In addition, the severity of the edema and their survival beyond e10.5 does not resonate with other knockout mouse models with established vascular permeability defects [39-41]. In contrast, the onset (*Calcrl*=E13.5, *Adm*=E14.5, *Ramp2*=E15.5) and severity of the phenotype closely resembles other genetic mouse models that delete genes essential for lymphatic development, including *Prox1 *[42], *Sox18* [43], and *Vegfc* [44].

To determine whether lymphatic vasculature defects may contribute to the edema observed in AM signaling KO animals, we performed a comprehensive study of AM signaling expression and function during lymphatic vascular development [22]. *Adm* is temporally and spatially expressed on the endothelium of the jugular vein in a polarized fashion towards the budding primary lymph sac *in vivo*, which is identical to the master lymphatic regulator, *Prox1* [42, 45, 46]. Moreover, *Calcrl* and *Ramp2* are preferentially up-regulated in LECs, partially under the control of the lymphatic-specific transcriptional regulator, Prox1. While loss of AM signaling did not affect the differentiation and migration of LECs to form the primary lymph sac or dermal lymphatics, it did lead to a hypoplastic jugular lymph sac due to decreased LEC proliferation. This result indicates that AM signaling is essential for normal LEC proliferation *in vivo *and the KO mice develop edema due to smaller jugular lymphatic vessels that are unable to maintain homeostatic fluid balance. It is also interesting to note that the jugular lymphatic trunk is affected by loss of AM signaling, while retroperitoneal and dermal lymphatic vessels appear normal. This indicates that there are different cellular mechanisms regulating different lymphatic beds during development. However, AM does appear to be an essential growth factor for developing LECs *in vivo *[22]. Thus, it is most likely that a combination of both blood and lymphatic defects leads to the edema and lethality in the KO mice given the integrated physiology between the two vasculatures. However, more specialized genetic assays are required to resolve the relative contributions of each vasculature within these KO mice [47]. 

An alternative approach to assess the role of AM signaling in development would be to use transgenic mouse models that overexpress *Adm*, *Calcrl*, or *Ramp2*. Interestingly, no developmental phenotypes have been reported in gain-of-function mouse models of AM signaling, either by vascular *Adm* overexpression [48] or vSMC-specific *Ramp2* overexpression [49], though these models displayed adult cardiovascular phenotypes. Given the essential nature of AM signaling within the endothelium, it would be interesting to over express *Calcrl* or *Ramp2* specifically in the endothelium, which to our knowledge, has not yet been reported.

### Adrenomedullin vs. Proadrenomedullin

One potential caveat with the majority of *Adm*^-/-^ studies is that the gene targeting strategies delete the entire *Adm* coding sequence [30, 31], which results in the genetic KO of two functionally active peptides, AM and proadrenomedullin N-terminal 20 amino acid peptide (PAMP) [50]. PAMP is a small peptide that is produced during post-transcriptional splicing of preproadrenomedullin and has numerous actions to complement or antagonize AM signaling [50-53]. For two of the reported *Adm* deficient mouse lines, the design of the targeted allele could not rule out whether the observed phenotypes in the KO animals were due to loss of AM, PAMP, or both [30, 31]. This controversy was partially resolved using a third independent *Adm*^-/-^ mouse, which left PAMP intact, and illustrated that loss of AM alone was enough to recapitulate embryonic lethality [32]. However, these mice lacking only AM had a milder phenotype (less edema and no cardiovascular abnormalities) when compared to KO mice lacking both peptides. This inconsistency in phenotypes could be attributed to differences in mouse strain and/or gene targeting approach [32]. However, a more intriguing hypothesis, which remains to be vigorously experimentally addressed, is that AM and PAMP may have non-redundant functions during cardiovascular development [54].

### Developmental Role of RAMP2 vs. RAMP3

While *Ramp2*^-/- ^mice recapitulated the *Adm*^-/-^ and *Calcrl*^-/-^ phenotypes, it appears that RAMP3, another RAMP that associates with CLR and binds AM, is not essential for embryonic survival since *Ramp3*^-/-^ mice develop normally to adulthood. There also appears to be no functional redundancy between RAMP2 and RAMP3 in development, since there is no transcriptional compensatory mechanism of either RAMP in response to loss of the other [18, 34]. Although RAMP3 has been implicated in receptor trafficking [7, 55, 56], the functional role of the AM/CLR/RAMP3 signaling complex is not well understood *in vivo*. 

### New Developmental Insights of Adrenomedullin Pathway

A recent study by Nicoli *et al.* expanded our knowledge regarding the role of CLR during embryonic vascular development using a zebrafish model. By knocking down *crlr* they showed drastic vascular defects due to decreased expression of *vegf*. While *vegf* appears to be the critical mediator in the vascular development since overexpression of *vegf* is able to rescue the *crlr* knockdown phenotype, it still appears that *crlr* is essential for appropriate levels of *vegf*. This study provides *in vivo* evidence that *crlr* is downstream of *sonic hedgehog*, but upstream of *vegf* and *notch* signaling in arterial differentiation and development [57]. Modulation of *vegf *levels by AM signaling were previously reported in mice [58] but a complete characterization of AM and VEGF interactions is not well understood. It is novel that *sonic hedgehog* appears to regulate *crlr* expression and further dissection of this pathway in animal models would improve our understanding of how CLR is regulated during development. The zebrafish model system has recently been used to study lymphatic development [59-61] and it would be interesting if phenotypes seen in *Adm*^-/-^, *Calcrl*^-/-^, and *Ramp*2^-/-^ mice could be recapitulated in zebrafish.

## PHYSIOLOGY AND PATHOLOGY

### Adrenomedullin Signaling in Pregnancy

AM signaling is known to be a critical component for initiation and progression of normal pregnancy. By the third trimester of a normal pregnancy, plasma levels of AM increase 4- to 5-fold [62-67]. AM is highly expressed in all vascular tissues which include the placenta and uterus [68-69]. Our previous studies in *Adm^+/- ^*female mice expressing 50% less adrenomedullin revealed that there is disrupted fertility, placentation, uterine receptivity, and fetal growth resulting from reduced AM expression [70]. AM signaling components are also expressed in the trophoblast cells [71-76], which take on an endothelial-like function during the process of decidual maternal spiral artery remodeling during pregnancy. The trophoblast giant cells deriving from the trophectoderm invade and replace the vascular wall by inducing a loss of endothelial cells and smooth muscle cell coverage to allow for higher blood flow to the fetus through the spiral arteries. Failure of this remodeling process to occur is a hallmark feature of pre-eclampsia. Further research needs to be performed to determine the extent to which AM signaling affects trophoblast cells in the process of maternal spiral artery remodeling during pregnancy. 

### Adrenomedullin Signaling and Cardiovascular Biology

AM has been reported to be upregulated in various cardiovascular conditions [1, 77, 78] and is a potent angiogenic factor as well as a cardioprotective factor [1]. Plasma AM increases 2-fold in conditions such as essential hypertension, renal failure and congestive heart failure [79, 80] (Fig. **1**). Previous studies with gene-targeted KO mice for *Adm *and *Calcrl *indicated that AM signaling is important for cardiovascular development [22, 30, 33]. Genetic reduction of *Adm* results in enhanced cardiovascular damage including increased cardiac hypertrophy in male *Adm^+/- ^*mice [81] and marked perivascular fibrosis, coronary artery intimal hyperplasia and oxidative stress with AngII/high-salt treatment [32]. AM protects the heart from hypertrophy and fibrosis during cardiovascular stress such as hypertension and cardiac hypertrophy, myocardial infarction, heart failure and atherosclerosis [82, 83], but the exact mechanisms of AM-mediated cardioprotection have not been fully elucidated. A comprehensive review of the cardioprotective function of AM during hypertension and heart failure has recently been provided by several groups [9, 84].

Endothelial dysfunction is characterized by reduced endothelium-dependent vascular relaxation which is associated with most forms of cardiovascular disease. It is partially impacted by reduced nitric oxide and upregulation of adhesion molecules to result in a proinflammatory and prothrombotic state [85]. Research also suggests that endothelial dysfunction may act as an early marker of atherosclerosis [86]. One study indicated that *Adm* and its receptor components, *Calcrl* and *Ramps* were upregulated in the aorta of apolipoprotein E-deficient (*ApoE*^-/-^) mice [87]. Loss of apoE ultimately results in a mouse model of spontaneous atherosclerosis because apoE is important in the removal of circulating lipoproteins [88]. When these mice were fed an atherogenic diet and treated with AM, the appearance of atherosclerotic lesions was reduced [87]. This study further indicates that AM may help to protect against the progression of atherosclerosis, but the exact mechanism for this action remains to be understood. Expression of adhesion molecules in LECs [89] as well as liver sinusoidal endothelial cells [90] were reduced in response to AM treatment. Similar results were seen with VEGF-treated HUVECs [91]. Thus, AM may impact endothelial dysfunction partially by modulating adhesion molecule expression. With respect to endothelium-dependent vascular relaxation, AM is known to induce vasodilation which is mediated partially by endothelium-derived nitric oxide [92-96]. Also, in a rat model of sepsis induced by cecal ligation and puncture, administration of AM and AM-binding protein (AMBP-1 also known as complement factor H) were shown to prevent against endothelial cell dysfunction and decreased endothelium-dependent vascular relaxation in thoracic aorta [97]. These studies implicate AM as having a protective role in cardiovascular disease and endothelial dysfunction, but further research needs to be performed to investigate how AM directly impacts cardiac endothelial cells to regulate their function. 

### The Role of Adrenomedullin Signaling in Response to Injury, Vascular Dysfunction and Wound Healing

Endothelial proliferation and angiogenesis are known to be impacted by AM signaling. In a hind-limb ischemia model, AM promotes endothelial cell proliferation and capillary formation and conversely, *Adm^+/- ^*mice showed reduced blood flow and capillary development [58]. Other whole animal studies using matrigel plugs demonstrated the role of AM in vascular regeneration because AM increased blood flow and capillary densities through PKA- and PI3K-dependent pathways [12, 13]. AM also induced tube-formation of HUVECs cultured on matrigel [14]. Another study pertaining to RAMP2 expression also revealed similar findings. An aortic ring assay and matrigel plug assay with adult *Ramp2^+/- ^*mice revealed that with decreased *Ramp2 *expression there was reduced neovascularization in response to growth factor stimulation [18]. Collectively, these studies indicate the importance of AM in endothelial cell proliferation and angiogenesis in adult mice. 

AM signaling is known to impact the blood and lymphatic vasculature in other physiological processes and pathological conditions. In a pathological mouse model of subcortical vascular dementia (chronic cerebral hypofusion), AM was shown to promote arteriogenesis and angiogenesis as well as inhibit oxidative stress and preserve white matter in the brain [98]. AM signaling can also induce anti-apoptotic and anti-inflammatory effects in response to injury. In the sinusoidal endothelial cells of the liver, AM helps to protect these cells from cold injury during the process of cold preservation for a liver transplant by decreasing endothelial cell apoptosis and inflammation [90]. Conversely, in *Adm^+/-^* and *Ramp2^+/^*^- ^mice there is increased apoptosis of the sinusoidal endothelial cells in the liver after cold injury [90] further indicating that AM signaling helps to regulate apoptosis. Wound healing is an essential physiological process that requires angiogenesis and lymphangiogenesis for proper healing. Since AM is a known angiogenic factor and lymphangiogenic factor [22], it is not surprising that AM signaling is necessary in the wound healing process. In an ischemia/reperfusion mouse model of a pressure ulcer, AM administration reduced the wound area and accelerated angiogenesis as well as lymphangiogenesis [99]. Also in a wounded HUVEC monolayer, AM promoted vascular regeneration *via *activation of endothelial Akt in a PKA- PI3K- dependent manner [12]. Lymphedema is a hallmark condition of lymphatic dysfunction resulting in the swelling of one or more limbs due to accumulation of interstitial fluid. In Balb/C mice with tail lymphedema, AM treatment improved lymphedema and increased the number of lymphatic and blood vessels near the injury site [11]. Taken together, these data indicate that AM is an essential component for proper endothelial cell function in both physiological and pathological states to regulate apoptosis, inflammation, and lymphangiogenesis as well as angiogenesis. 

An important issue to still address is to determine the exact role of AM signaling during adulthood by using temporal and spatial KO mice for components of the AM signaling system to evaluate physiology and function of the vascular beds in these mice. Previous studies with genetic KO mice for the AM signaling system reveal an enhanced impact of AM on lymphatic vascular development relative to blood vascular development [22]. It has also been shown that the gene expression of AM receptor components, *Calcrl *and *Ramp2,* are enhanced in LECs compared to BECs [23, 24]. Due to these known differences of AM signaling between BECs and LECs, it would be interesting to determine whether there is also an enhanced effect of AM on the lymphatic vasculature in adult physiology and pathology. The underlying mechanisms through which AM impacts the lymphatic vasculature, blood vasculature as well as the more specialized cardiac tissue during adulthood also needs to be identified. 

### Adrenomedullin Expression in Tumor Progression

The AM peptide was initially isolated from a human adrenal tumor (pheochromocytoma) due to its platelet cAMP elevating activity [78]. Since this discovery almost 20 years ago, investigation into the role of AM in tumors has greatly expanded. Early studies noticed elevated levels of AM in lung and brain tumors [100, 101] and a comprehensive survey of human tumor cell lines from lung, breast, brain, ovary, colon, and prostate substantiated those reports [102]. AM has been implicated in a variety of pro-tumor functions including acting as an autocrine growth factor [102-104], apoptosis survival factor [15], promoter of tumor cell motility and invasion [104-106], and molecular intermediate to enhance communication between tumor cells and immune cell infiltrates [107]. Furthermore, it has been suggested that the presence of AM in tumors may signify a more aggressive tumor phenotype due to correlation between *Adm* gene expression and histological tumor grade [104, 108]. 

The mechanism(s) by which *Adm* gene expression is transcriptionally regulated in tumors remains unclear. It is likely that AM can be both an autocrine and paracrine factor [109] by providing tumor cells a growth advantage in addition to acting on surrounding endothelial cells to promote proliferation and changes in vessel permeability to perhaps facilitate metastasis. Moreover, it has been suggested that hypoxia may play a role in AM production [8, 110]. Tumors often develop hypoxic zones in areas where blood flow is inadequate to supply the necessary oxygen required for the growing tumor cells. As a result of this unfavorable state, hypoxia inducible factor-1 (HIF-1) is activated which in turn upregulates a number of genes to compensate for the reduced oxygen microenvironment. Interestingly, a HIF-1 dependent mechanism was found to increase the expression of *Adm* in hypoxic human tumor cell lines [111]. Furthermore, *Adm* and *Calcrl* were found to be upregulated in microvascular endothelial cells cultured under low oxygen conditions [112]. Together, these results show that both tumor cells and surrounding endothelial cells are responsive to hypoxic conditions and may provide a mechanism for elevated AM levels in a tumor setting. 

Although the precise role of AM in tumor development and progression is still unresolved, significant progress has been made to better understand how AM affects not only a tumor cell, but also the endothelial cells in the surrounding microenvironment. Analysis of immunohistochemical staining of human ovarian cancer found that in addition to tumor cells, AM was also localized to the endothelial cells of the surrounding stroma [108]. Furthermore, an *in vitro* co-culture system found that HUVECs became activated upon exposure to tumor cells and consequently increased transcriptional activity of *Adm*, among other factors [113]. Since AM directly impacts endothelial cell proliferation and permeability, AM induced modulation of vessels may affect the spread of cancer cells to distant sites *via *blood or lymphatic vasculature. Research groups have been performing the *in vivo* studies necessary to confirm that AM promotes tumor progression through its known angiogenic properties. Several reports have shown that inhibition of AM action by neutralizing antibodies or AM antagonist AM_22-52_ have reduced the growth of tumor xenografts *in vivo* by suppressing vascular development [58, 114, 115]. 

While much of the focus in understanding the process of tumor (lymph)angiogenesis has been upon the VEGF protein family, the contribution of AM to this process should not be underappreciated. Clearly, the studies described above point to AM as a valid target for potential cancer therapies although more research is necessary. Generation and validation of preclinical mouse models that are able to rigorously test AM as a target are greatly needed. Since the embryonic lethal phenotype of *Adm*^-/-^ mice makes studying this signaling pathway more complicated, novel genetic mouse models (Table **1**) using conditional alleles [18, 22, 116] and vascular endothelium specific *Cre* animals are a starting point for such tumor studies. Furthermore, these mouse models will be needed to refine our understanding of the metastatic process. Given the knowledge that AM can act on both the blood and lymphatic endothelium, a key question that remains to be answered is by what mechanisms do tumor cells disseminate into the blood and/or lymphatic vessels. 

## SUMMARY AND FUTURE DIRECTIONS

**Fig. (2):**
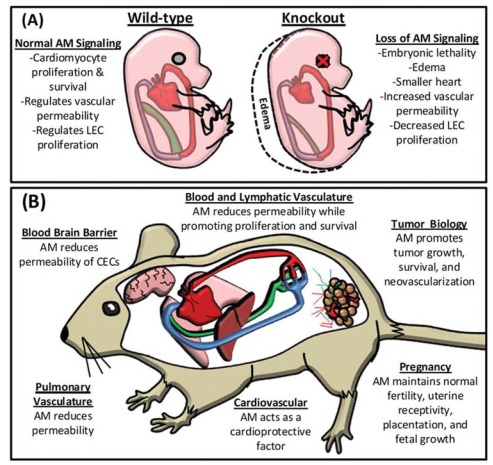
**Adrenomedullin Signaling in Development and Vascular Biology.** (**A**.) Loss of AM signaling causes embryonic lethality due to severe edema associated with impaired lymphatic vascular development. (**B**.) In the adult, AM is an angiogenic, lymphangiogenic, and a cardioprotective factor that also regulates vascular permeability and inflammation. Expression of AM is also implicated in pregnancy and tumor progression.

The use of genetic animal models in the field of AM research has produced significant contributions toward understanding the biology of this pleiotropic molecule, with a renewed appreciation for it's critical regulation of endothelial cell function during development and vascular diseases. To date, AM has been implicated in lymphatic vascular development, in proper functioning of blood and lymphatic endothelial cells and in a variety of conditions such as pregnancy, cardiovascular disease, and tumor progression (Fig. **2**). Despite the strides that have been made, there is much more to learn regarding the mechanisms mediating AM function and regulation. With the generation of additional sophisticated molecular biology tools such as genetic mouse models, we are poised to refine our current knowledge as well as discover other novel roles for this peptide and signaling partners in normal and disease physiology.

## Figures and Tables

**Table 1.** Gene Targeted Mouse Models for Studying Adrenomedullin Signaling

Mouse Model	Development or Adulthood	Result	Reference
*Adm^-/-^*	Development	-Embryonic lethal (e14.5), edema, smaller hearts, reduced myocyte proliferation and increased apoptosis, increased left ventricle trabecularization, thinner aorta and carotid artery walls, increased vascular permeability, hypoplastic jugular lymph sac	[22,30-32]
*Adm^+/-^*	Adulthood	-Pregnancy: Disrupted fertility, placentation, and fetal growth -Cardiovascular: Increased damage including hypertrophy, reactive oxygen species (ROS), and fibrosis -Liver cold injury: increased apoptosis of the sinusoidal endothelial cells	[32,70,81,90]
*Adm^fl/fl^/Tubulin Tα-1-Cre^+^*	Adulthood	-High anxiety, hyeractive, impaired motor coordination	[116]
*Calcrl^-/-^*	Development	-Embryonic lethal (e13.5), similar phenotype as *Adm^-/- ^*mice	[22,33]
*Calcrl^LoxP/-^/Tie2Cre^+^*	Development	-Embryonic lethal (e16.5) and recapitulation of *Adm^-/-^*, *Calcrl^-/-^*, and *Ramp2^-/-^*phenotype	[22]
*Ramp2^-/-^* and *Ramp2^fl/fl^/CAG-Cre^+^*	Development	-Embryonic lethal (e15.5), similar phenotype as *Adm^-/- ^*mice	[18,22,34]
*Ramp2^+/-^*	Adulthood	-Increased vascular permeability and decreased neovascularization -Liver cold injury: increased apoptosis of the sinusoidal endothelial cells	[18,90]
*PAM^-/-^*	Development	-Embryonic lethal (e14.5) and phenocopy of *Adm^-/-^*, * Calcrl^-/-^*, and *Ramp2^-/-^*mice due to loss of amidation of AM peptide	[35]

Adrenomedullin (Adm); Calcitonin receptor-like receptor (Calcrl); Receptor activity modifying protein (RAMP); Peptidylglycine alpha-amidating monooxygenase (PAM)

**Table 2.** Vascular Assays for Studying Adrenomedullin Function

Assay	Result	Reference
Atherogenic Model	-Atherogenic diet and AM treatment in *ApoE^-/-^*mice resulted in reduced formation of atherosclerotic lesions	[87]
Tail microlymphography	-AM injected mice showed reduced permeability of the dermal lymphatic capillaries	[25]
Matrigel plug	-AM increased vascular regeneration -*Ramp2^+/-^* mice exhibited reduced neovascularization	[12,13,18]
Aortic ring	*-Ramp2^+/-^*mice exhibited reduced neovascularization in response to growth factor stimulation	[18]
AngII/high-salt	AM^+/-^ mice exhibited increased reactive oxygen species (ROS), vascular fibrosis, and intimal thickening	[39]
Prolonged mechanical ventilation	-AM treatment reduced lung vascular permeability resulting from ventilator use	[21]
Chronic cerebral hypofusion	-AM promoted arteriogenesis and angiogenesis	[98]
Hind-limb ischemia	-AM promotes endothelial cell proliferation and capillary formation -*Adm^+/-^* mice showed reduced blood flow and capillary development	[58]
Wound healing (Pressure Ulcer -Ischemia reperfusion model)	-AM reduced wound area and increased angiogenesis and lymphangiogenesis	[99]
Tail lymphedema	-AM improved lymphedema and increased number of lymph and blood vessels	[11]
Tumor xenografts	-Blocking AM signaling results in reduced vascular development	[58, 114, 115]
